# A 56-Year-Old Woman with Chronic Hepatitis C Liver Disease and Meningitis due to *Streptococcus equi* subsp. *Zooepidemicus*

**DOI:** 10.1155/2021/7227054

**Published:** 2021-10-01

**Authors:** Sebastian Klapa, Jochen Grefer, Ingo Sobottka, Volkhard Kurowski

**Affiliations:** ^1^DRK-Hospital Moelln-Ratzeburg, Ratzeburg, Germany; ^2^Section of Maritime Medicine, Institute of Experimental Medicine, Christian-Albrechts-University Kiel c/o German Naval Medical Institute, Kronshagen, Germany; ^3^LADR Central Laboratory MVZ Dr. Kramer and Colleagues, Geesthacht, Germany

## Abstract

*Streptococcus equi* subsp. *zooepidemicus* (*S. equi* subsp. *zooepidemicus*), which carries the Lancefield group C antigen, is an uncommon human pathogen. It is considered an opportunistic commensal of the equine upper respiratory tract and causes invasive infections in immunocompromised hosts, following close contact to infected horses. Meningitis caused by *S. equi* subsp. *zooepidemicus* is a rare infectious disease with high rates of complications. We present the case of a 56-year-old female with acutely altered mental status following three days of fever and vomiting. For several months, she was taking care of horses. The most relevant preexisting illnesses were chronic hepatitis C infection and traumatic paraplegia due to spinal cord injury 30 years ago. Laboratory evaluation on admission revealed leukocytosis, hyponatremia, and elevated C-reactive protein. Cerebral CT scan showed diffuse cerebral edema. Whereas cerebrospinal fluid real-time PCR assay for common pathogens was negative, cultures showed *S. equi* subsp. *zooepidemicus*. She recovered fully after intravenous administration of ceftriaxone for four weeks. This is one of only few reported cases of *S. equi* subsp. *zooepidemicus* meningitis and the first case in chronic hepatitis C infection. Our case supports the necessity for extended microbiological examination especially in immunocompromised patients if PCR examination for common pathogens is negative.

## 1. Introduction

*Streptococcus equi* subsp. *zooepidemicus* (*S. equi* subsp. *zooepidemicus*) belongs to a group of beta-hemolytic *streptococci* with Lancefield group C antigen, whereas *S. dysgalactiae* subsp. dysgalactiae, *S. dysgalactiae* subsp. equisimilis, *S. equi* subsp. equi, *S. anginosus*, *S. constellatus* subsp. pharyngis, and *S. phocae* may contain the C antigen as well [1]. In contrast to *S. equi* subsp. *equi*, *S. equi* subsp. *zooepidemicus* is a typically opportunistic pathogen in horses and causes severe diseases such as pneumonia, arthritis, and wound infection [1]. In humans, *S. equi* subsp. *zooepidemicus* infections are rare but capable to induce a variety of clinical symptoms including sinusitis, endocarditis, septic arthritis, osteomyelitis, pericarditis, and streptococcal toxic shock syndrome [2]. However, there are only a few reported cases of meningitis, especially in immunocompromised patients, with an increased risk of complications [3, 4]. We report a case of meningitis caused by *S. equi* subsp. *zooepidemicus* in a patient with chronic hepatitis C. This has previously not been recognized as an infectious agent in this context.

## 2. Case Report

**Figure 1:**
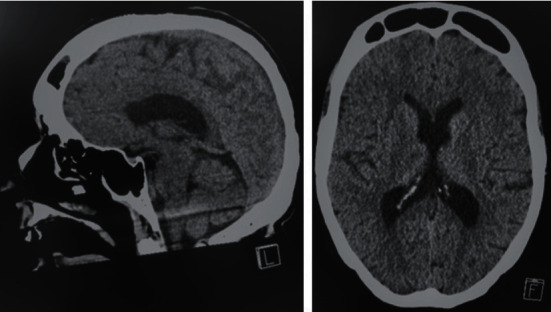
CT brain image obtained in the emergency department indicating a diffuse cerebral edema consistent with meningoencephalitis.

**Figure 2:**
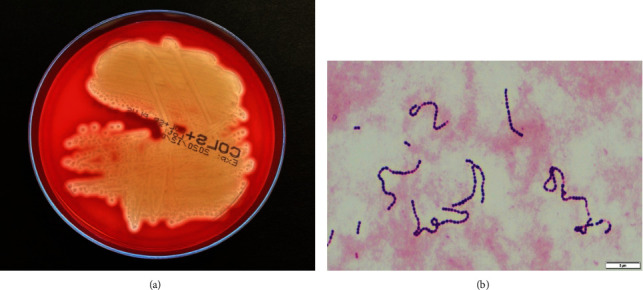
(a) Cerebrospinal fluid cultures revealed *β*-hemolytic *streptococci* that could be identified as *S. equi* spp. *zooepidemicus*. (b) Gram staining of the cerebrospinal fluid culture showing gram-positive cocci.

A 56-year-old Caucasian female presented with a 3-day history of fever, vomiting for 2 days, and altered sensorium since the morning of presentation to our emergency department. On examination, the weight was 52 kg and the height was 168 cm. She suffered from complete paraplegia due to spinal cord injury 30 years ago with absence of motor and sensory function below Th1 and from chronic hepatitis C infection. She was drowsy but arousable. At the time of admission, body temperature was 101.8 F, with otherwise normal vital signs. Except for preexisting paraplegia, there were no further symptoms or signs of neurological deficit, in particular, no nuchal rigidity. Laboratory chemistry revealed normal hepatic and altered inflammatory parameters (total bilirubin of 0.68 mg/dl, alkaline phosphatase of 102 U/l, aspartate aminotransferase of 87 U/l, alanine aminotransferase of 24 U/l, leukocyte level of 19 G/l, glucose of 147 mg/dl, and high-sensitivity C-reactive protein of 153 mg/l), as well as reduced levels of natrium (131 mmol/l). Initial computed tomography scan of the brain showed diffuse cerebral edema (Figure 1). The patient was admitted to our intensive care unit, where due to suspected meningoencephalitis intravenous administration of ceftriaxone, ampicillin, dexamethasone, and aciclovir was started. Cerebrospinal fluid (CSF) examination was performed shortly after first doses of antibiotic therapy. The CSF was turbid, with increased protein of 889.6 mg/dl, reduced glucose of 19 mg/dl, and elevated neutrophil granulocytes of 3478 cells/*μ*l. CSF real-time PCR assay for common CSF pathogens (*Streptococcus agalactiae*, *Escherichia coli* K1, *Haemophilus influenzae*, *Listeria monocytogenes*, *Neisseria meningitides*, *Streptococcus pneumoniae*, HSV 1/2, VZV, CMV, and *Cryptococcus neoformans*/*gattii*) was negative. Blood cultures taken on admission were negative. CSF cultures confirmed *β*-hemolytic, gram-positive *streptococci* (Figure 2) which were positive for Lancefield group C that could subsequently be identified as *S. equi* subsp. *zooepidemicus* by mass spectrometry due to MALDI-TOF MS (Bruker®) using the MBT IVD Library 9.0 giving a score value of 2.49. In addition, the cerebrospinal fluid was examined by a commercial PCR targeting the 16S ribosomal DNA (SepsiTest™-UMD; Molzym, Bremen, Germany). DNA was extracted by MolYsis™ complete kit (Molzym). Both kits were used according to the manufacturer's instructions. The PCR product of 430 base pairs was sequenced and used for Blast® search in GenBank® (NCBI, NIH, Bethesda MD, USA). The sequence obtained from the CSF was identical to *S. equi*, but subspecies could not be identified by this molecular tool.

The *S. equi* subsp. *zooepidemicus* isolates were tested susceptible for ceftriaxone as well as for penicillin and ampicillin. Resistance determination was carried out according to the current EUCAST Standard Version 11.0 of 2021 using agar diffusion methods. Searching for a possible path of infection, a small chronic wound on the left leg was observed. However, multiple cultures of the skin lesions were tested negative. The patient confirmed close contact to horses before onset of symptoms and recovered completely after a two-week course of ceftriaxone.

## 3. Discussion

*S. equi* subsp. *zooepidemicus* is known to be pathogenic especially in horses and considered as an opportunistic commensal of the upper respiratory tract. In humans, contact with infected horses or dogs or ingestion of unpasteurized cheese or milk from infected cows has reported prerequisites for infection with *S. equi* subsp. *zooepidemicus* [4]. In our patient, the most probable source of infection was a preceding close contact with a presumably infected horse. Literature search using the terms “*Streptococcus equi*” in combination with “meningitis” evaluated 32 cases of reported meningitis with *S. equi* subsp. *zooepidemicus* [2–5]. Classic clinical symptoms of meningitis were present in nearly half of the patients [5]. As in our case, the clinical presentation may be subacute. Our patient did not demonstrate typical meningitis symptoms like headache, photophobia, or neck stiffness. This can lead to misdiagnosis and delay of appropriate therapy.

Moreover, the real incidence of *S. equi* subsp. *zooepidemicus* meningitis may be higher as expected. Most of the regularly used real-time PCR tests for common CSF pathogens do not include the detection of *S. equi* subsp. *zooepidemicus*. These *streptococci* usually respond to treatment with *β*-lactam antibiotics, which are recommended and used for therapy of unclear pathogens [4].

Immunosuppression may be a risk factor for infection with *S. equi* subsp. *zooepidemicus* [3, 4]. Our patient suffered from chronic hepatitis C infection without current need of antiviral treatment. In contrast to a published case of a hepatitis C patient with primary *S. equi* subsp. *zooepidemicus* bacteremia, our patient did not develop liver cirrhosis [6]. However, chronic hepatitis C may also induce a functional impairment of CD4+ as well as CD8+ T cells resulting in an impaired immune response [7, 8].

Fortunately, our patient recovered completely following a two-week administration of intravenous ceftriaxone. Remarkably, there is a crude mortality rate of 24% in patients with *S. equi* subsp. *zooepidemicus* meningitis, and hearing loss is a frequent complication (19%). Only 38% of patients recover completely; the majority of recovered patients received extended therapy with penicillins or third-generation cephalosporins [9].

## 4. Conclusion

In particular, in cases of unclear pathogens in sepsis and/or meningitis, multiple cultures of relevant samples for microbiological diagnostic before antibiotic treatment are highly important.
